# Supplementary material for: Network Analysis Provides Insight into Tomato Lipid Metabolism
Source: Metabolites. 2020 Apr 14;10(4):152. doi: 10.3390/metabo10040152 (PMC7240963; doi:10.3390/metabo10040152)
Supplement: Supplementary file 1 [file metabolites-10-00152-s001.zip › metabolites-10-00152-s001/supplementary_figures_732979.pdf]

Article

Network analysis provides insight into tomato lipid metabolism

Anastasiya Kuhalskaya [1,2+], Micha Wijesingha Ahchige [1+], Leonardo Perez de Souza [1], Jose Vallarino [1], Yariv Brotman [1,2] and Saleh Alseekh [1,3*]

Supplementary Material

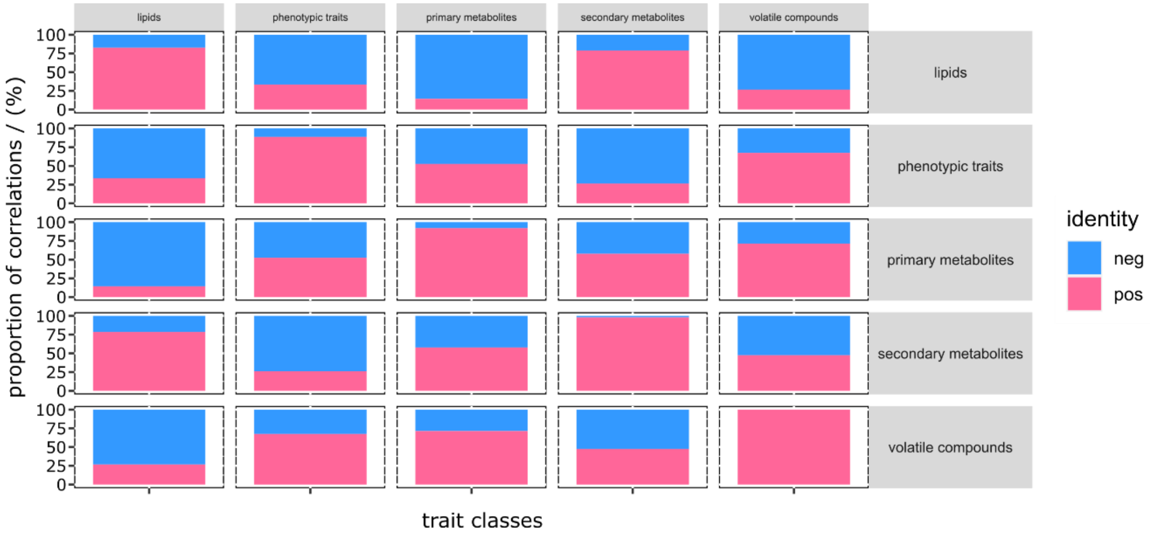

Figure S1. Percentage of positive and negative correlations in overall metabolic network. Majority of correlations in the network were positive (84.4%). Within the same group of metabolites or phenotypic traits, connections were predominantly positive. High amount of negative correlations were occurred between lipids and phenotypic traits, volatiles, primary metabolites; between phenotypic traits, volatiles and specialized metabolites.

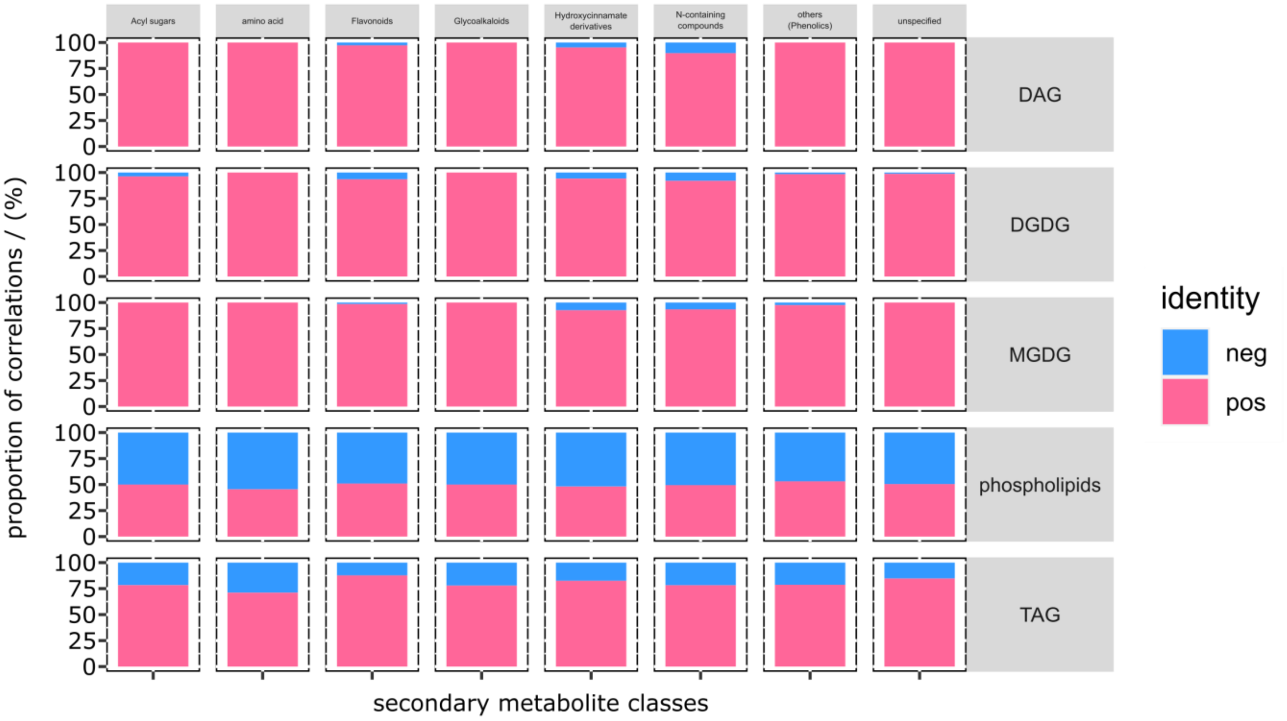

Figure S2. Percentage of positive and negative correlations between lipophilic compounds and secondary metabolites. Galactolipids and DAGs showed mostly positive correlations with all classes of specialized metabolites. In comparison, phospholipids and TAGs displayed slightly higher amount of negative connections with secondary metabolites.

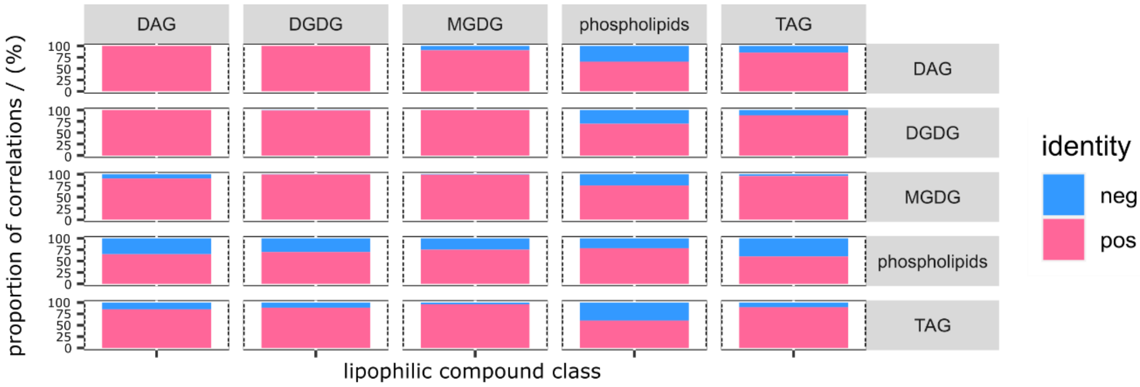

Figure S3. Percentage of positive and negative correlations within the class of lipophilic compounds. All lipids belonging to the same subclass showed mainly positive connections with other members of the same class. Galactolipids correlates to each other predominantly positively. There are no negative correlations between DAGs and DGDGs. The highest percentage of negative correlations was observed between phospholipids and TAGs.
